# Submacular Injection of Ranibizumab as a New Surgical Treatment for Refractory Diabetic Macular Edema

**DOI:** 10.1155/2019/6274209

**Published:** 2019-10-21

**Authors:** Samir M. El-Baha, Ahmed M. Abdel Hadi, Mahmoud A. Abouhussein

**Affiliations:** Ophthalmology Department, Alexandria University, Alexandria, Egypt

## Abstract

**Purpose:**

In this study, we describe a new surgical technique for the treatment of refractory DME. The technique consists of vitrectomy with ILM peeling with a subretinal injection of ranibizumab.

**Methods:**

This is a prospective interventional noncomparative study including patients with refractory DME. Included patients were subjected to the new surgical technique of pars plana vitrectomy with subretinal injection of ranibizumab.

**Results:**

The study included 19 eyes with refractory macular edema, in which this novel technique was attempted. There were 10 males and 9 females. The age of the patients ranged from 17 to 67 years with a mean of 55.58 ± 13.242 years. The duration of diabetes before enrollment in the study ranged from 7 to 25 years with a mean of 16.3 years. Preoperatively, the mean CMT of the eyes ranged from 352 to 883 microns with mean ± SD of 498.58 ± 152.16 microns. Postoperatively, this improved significantly to 373.5 ± 100.3, 355.9 ± 89.8, and 365.74 ± 120.12 microns at 1, 3, and 6 months, respectively (*p* ≤ 0.001 for all).

**Conclusion:**

This novel surgical procedure of vitrectomy with ILM peeling with a subretinal injection of ranibizumab is effective in cases of refractory DME. The study has been registered in Contact ClinicalTrials.gov PRS Identifier: NCT03975088.

## 1. Introduction

Diabetic macular edema (DME) is the main cause of visual loss in patients with diabetic retinopathy [1].

Macular laser photocoagulation was the main treatment for DME according to the Early Treatment Diabetic Retinopathy Study (ETDRS) [2]. It remained the gold standard management until the availability of intravitreal anti-VEGFs which proved to be an effective treatment option for DME [3]. Intravitreal anti-VEGFs injections showed better functional and anatomic outcomes than macular laser photocoagulation [4]. Nevertheless, patients with DME need repeated multiple injections over a long period of time leading to a problem with compliance. Also, patients with diabetes and other cardiovascular diseases might not tolerate repeated intravitreal anti-VEGFs injections [3, 4].

Furthermore, despite the benefit of anti-VEGF injections, DME can persist in some patients [5]. The incidence of patients with persistent DME is about 40–50% after receiving monthly ranibizumab [5–7].

The problem with refractory persistent DME is the irreversible vision loss that results from permanent photoreceptor damage [8]. Even if delayed treatment is given and the edema resolves, the functional outcome will be unsatisfactory due to retinal architecture damage. In the three-year report of the RISE and RIDE study, patients who received sham treatment for the first 2 years and then were switched to monthly ranibizumab showed good anatomic results with less visual gain compared to the group on monthly ranibizumab from the start [9].

The best treatment strategy for refractory DME is not known. Options include switching between anti-VEGF agents, corticosteroids, a combination of anti-VEGF and corticosteroids, and vitrectomy.

Vitrectomy for DME is a less expensive option compared to repeated intravitreal injections of anti-VEGFs [10]. Its beneficial effect was reported for both tractional DME and nontractional refractory DME [11].

The Diabetic Retinopathy Clinical Research (DRCR) Network showed the good outcome of vitrectomy for tractional DME. This was reported with good visual and anatomic results [12].

The role of vitrectomy for refractory nontractional DME was also reported. Vitrectomy allows a more efficient clearance of VEGF and other mediators from the retina, leading to more oxygen availability for the retina reducing the edema [13, 14]. Also, the vitreous samples from diabetic patients show increased collagen crosslinking [15], keeping high VEGF levels near the retinal surface [16]. Vitrectomy also removes cellular mediators and growth factors that might be the cause of treatment-resistant DME [17, 18].

ILM peeling might contribute to diabetic macular edema treatment by reducing tangential traction, thus eliminating the scaffold that helps the growth of astrocytes decreasing the possibility of epiretinal membrane formation after surgery [19].

One of the problems faced after vitrectomy for diffuse DME is that the resolution of macular edema is not always associated with a similar functional improvement [12]. In contrast with the rapid resolution of edema after intravitreal anti-VEGFs, edema is reduced more gradually over a longer period of time after vitrectomy. The presence of chronic edema can cause irreversible photoreceptor damage and bad visual outcome [20–24].

In a trial to overcome this problem, Morizane et al. [25] reported their technique of intended macular detachment for the rapid treatment of diffuse DME. They showed that subretinal BSS injections after vitrectomy with ILM peeling might be a useful technique for rapidly treating DME. The benefit was shown in naïve cases and refractory cases.

Subretinal injection of anti-VEGFs was reported in several studies for the treatment of submacular haemorrhage due to exudative age-related macular degeneration [26, 27].

The subretinal space, by definition, is the space between RPE cells and photoreceptors [28]. The effect of a drug delivered in the subretinal space might exceed the effect of the same drug injected intravitreally due to the closer contact with the retina without intervening barriers [28].

In this study, we describe a new surgical technique for the treatment of refractory nontractional DME. The technique consists of vitrectomy with ILM peeling with a subretinal injection of ranibizumab.

## 2. Patients and Methods

This is a prospective interventional noncomparative study including patients with DME resistant to anti-VEGF injections. Included patients were enrolled between June 2016 and June 2017; they were subjected to the new surgical technique of pars plana vitrectomy with subretinal injection of ranibizumab.

The study followed the Declaration of Helsinki. All patients were informed about the details of the new surgical technique, and informed consent was obtained. The study was approved by the ethics committee of the university.

To be included in this study, patients must be diagnosed with refractory diffuse nontractional DME. Central retinal thickness (CRT) should exceed 350 *μ*m despite undergoing multiple anti-VEGF therapies. We defined refractory DME as eyes with persistent DME despite receiving at least 6 monthly ranibizumab injections of anti-VEGF and then switched to Aflibercept, receiving at least three monthly injections. Decimal best-corrected visual acuity (BCVA) must be ≥0.01 and ≤0.5.

Exclusion criteria included previous vitrectomy, recent cataract surgery less than 6 months, evident RPE atrophy, active proliferative diabetic retinopathy, massive foveal hard exudation, foveal traction on OCT, glaucoma, and one-eyed patients.

All patients underwent complete ophthalmologic examinations with special emphasis on best-corrected visual acuity (BCVA) using the 6 m Landolt C acuity chart (converted to decimal visual acuity) and indirect and contact lens slit-lamp biomicroscopy. Fluorescein angiography was done using Topcon fundus camera (Topcon Corporation, Tokyo, Japan). All eyes were examined by Spectral-Domain OCT (Spectralis; Heidelberg Engineering GmbH, Heidelberg, Germany). OCT scans were obtained preoperatively and at 1 month, 3 months, and 6 months postoperatively. All patients were followed up for at least 6 months.

## 3. Surgical Technique

The surgeries were performed using Constellation Vision System (Alcon, USA) 23-gauge system. After core vitrectomy, we stained the ILM with 0.25 mg/mL Coomassie brilliant blue G 250 solution (Sigma-Aldrich, St. Louis, MO) and removed the ILM. We then injected 0.5 mL of filtered air into the subretinal space to detach the fovea, ensuring that the foveal detachment covered the entire area of the DME. This injection of air was performed at the site where the ILM had been removed using a 38-gauge cannula (MedOne Surgical Inc., Sarasota, FL) with a pressure of 4 to 6 pounds per square inch (psi) (viscous fluid control system, Alcon Laboratories, Fort Worth, TX). The injected filtered air enters the subretinal space and causes foveal retinal detachment allowing easier subsequent injection of ranibizumab. Subretinal injection of 0.05 ml of ranibizumab (0.5 mg) was done using the same 38-gauge cannula (See Video in the supplementary materials which demonstrates our technique).

At the end of the procedure, partial fluid air exchange was done. After the surgery, a prone position was adopted for 24 hours.

The change in central retinal thickness and the change in best-corrected visual acuity were assessed at the 6-month follow-up visit. Surgical complications and any adverse events were recorded.

Data were analyzed using Statistical Package for Social Sciences (SPSS/version 23) software. *t*-test was used for comparison between different periods of follow-up with baseline. The level of significance was 0.05.

## 4. Results

The study included 19 eyes of 19 patients with refractory diabetic macular edema. There were 10 males and 9 females. The age of the patients ranged from 17 to 67 years with a mean of 55.58 ± 13.242 years. The duration of diabetes before enrollment in the study ranged from 7 to 25 years with a mean of 16.3 years. Preoperatively, all patients were pseudophakic. The patients were followed up from 6 to 7 months with a mean of 6.3 months.

Before surgery, OCT showed that 16 eyes (84.2%) had cystoid macular edema, 3 eyes (15.7%) had spongiform retinal edema, and 8 eyes (42.1%) had serous retinal detachment.

Preoperatively, the decimal best-corrected visual acuity ranged from 0.01 to 0.2 in decimal form with a mean of 0.068 ± 0.052. Postoperatively, this improved to a mean of 0.11 ± 0.05, 0.16 ± 0.09, and 0.2 ± 0.12 at 1, 3, and 6 months, respectively. All of these means were statistically better than the preoperative VA (*p* ≤ 0.001 for all time points compared to initial visual acuity).

At the end of follow-up, best-corrected visual acuity improved in 17 eyes (89.5%) and remained unchanged in 2 eyes (10.5%) in comparison to baseline best-corrected vision. There were no cases of visual acuity worsening.

Most of our patients (17 eyes, 89.5%) had preoperative visual acuity of 0.1 or lower. At the end of follow-up, only 7 eyes (36.8%) had a best-corrected vision of 0.1 or less.

Preoperatively, the mean CMT of the eyes ranged from 352 to 883 microns with mean ± SD of 498.58 ± 152.16 microns. Postoperatively, this improved significantly to 373.5 ± 100.3, 355.9 ± 89.8, and 365.74 ± 120.12 microns at 1, 3, and 6 months, respectively (*p* ≤ 0.001 for all). Moreover, the CMT dropped further from the first month to the third month; this was statistically significant (*p*=0.013). There was a subsequent increase of CMT from the third month to the sixth postoperative month, but this was not statistically significant (*p*=0.236).

Preoperatively, it was difficult to comment on the external limiting membrane (ELM) and the ellipsoid zone (EZ) due to attenuation of the OCT signals by the marked edema in most eyes. At the 6-month follow-up visit, EZ was intact in 11 eyes (57.9%) and disrupted in the rest. ELM was found to be intact in 13 eyes (68.4%).

Both the postoperative best-corrected visual acuity and central macular thickness (at 6 months) were statistically better than their preoperative counterparts regardless of the mean duration of diabetes or the mean age of the patients.

No surgery-related complications were recorded in any of the study eyes. No systemic side effects were observed in any of the patients during the follow-up period.

## 5. Discussion

The treatment of choice for patients with refractory DME, resistant to repeated intravitreal anti-VEGFs injections, is not yet determined.

This prospective interventional study describes a new surgical technique of vitrectomy with subretinal injection of ranibizumab for refractory nontractional DME. The results showed statistically significant improvement in both visual acuity and central retinal thickness at 6 months after surgery compared to the preoperative data.

The concept of using the subretinal space in retinal surgery was introduced for many purposes: genetic treatment of retinal degenerations [29], massive submacular haemorrhage [30], macular translocation in AMD [31], and macular hard exudates removal [32].

The choice of ranibizumab for the subretinal injection in this new surgical technique was based on its rapid systemic clearance. Ranibizumab molecule was designed without the Fragment constant (Fc) domain to allow for rapid systemic clearance, in contrast to Fc-containing molecules, such as Bevacizumab and Aflibercept, which have a much lower rate of systemic clearance [33]. In a study of exudative AMD patients receiving intravitreal anti-VEGFs injections, it was shown that ranibizumab has a minimal effect on plasma-free VEGF concentrations, whereas Bevacizumab and Aflibercept caused a significant reduction of the free VEGF due to greater systemic exposure [34].

The delivery of subretinal anti-VEGFs following pars plana vitrectomy was reported for massive subretinal haemorrhage due to neovascular age-related macular degeneration. One study used subretinal Bevacizumab [26]. The described technique used a 41-gauge extendable subretinal cannula (23 gauge; DORC) connected to a syringe which was inserted at the superior edge of the submacular haemorrhage and injected in the subretinal space. The injection consisted of a combination of 0.4 mL of recombinant tissue plasminogen activator (rtPA) at a concentration of 12.5 *μ*g/0.1 mL (total, 50 *μ*g), 0.1 mL of Bevacizumab (2.5 mg), and filtered air [26].

Another study used subretinal ranibizumab [27]. The mixture consisted of 0.5–0.7 ml of rtPA 125 *μ*g/ml and 0.06 ml of ranibizumab 10 mg/ml for submacular haemorrhages secondary to neovascular age-related macular degeneration [27].

Some studies reported that diabetic macular edema takes a long period of time to improve after vitrectomy [35, 36]. One study showed that only 50% of patients had a resolution of DME by 3 months, while another 47% of patients took one year to show improvement [35]. Since longstanding edema can result in permanent damage of photoreceptors, it is beneficial to try to shorten the duration of DME to maximize the visual gain. It was shown that the shorter the duration of DME before surgery, the better the postoperative ELM and EZ on OCT [37].

A new surgical technique for rapid resolution of DME was recently described. The surgical procedure was named planned foveal detachment technique for the resolution of diffuse diabetic macular edema [25]. The technique consists of vitrectomy with the use of triamcinolone acetonide to enhance the visualization of the posterior hyaloid. After core vitrectomy and ILM peeling, a small retinal detachment at the fovea was done by injecting a balanced saline solution under the macula. The reported results show rapid visual and anatomic improvement after this technique [25], but the possibility that residual triamcinolone acetonide contributed to the treatment of diabetic macular edema cannot be excluded.

The subretinal injection of ranibizumab after vitrectomy with ILM peeling has the potential advantage of accelerating the resolution of the macular edema after vitrectomy. Our results showed that this new surgical technique produced statistically significant improvement in visual acuity and central retinal thickness as early as 1 month after surgery.

All of the eyes included in this study were pseudophakic, so the recorded visual gain was attributed only to the new surgical technique with no influence from cataract removal.

Most of our patients (17 eyes, 89.5%) had preoperative decimal visual acuity of 0.1 or lower. In these cases, although there was a decrease in DME after surgery, the postoperative best-corrected visual acuity did not increase much probably due to chronic damage of the photoreceptor layer. Future studies are needed to confirm whether operating earlier on eyes with better initial visual acuity and less chronic edema is beneficial.

We noticed an increase in the mean central macular thickness at 6 months (365.74 microns) compared to the mean thickness at 3 months (355.9 microns). This may reflect that the beneficial effect of subretinal ranibizumab began to fade at this stage, indicating the short-term benefit of this procedure.

This study has some limitations, including the lack of a control group, a small number of patients, and a short duration of follow-up. Future randomized controlled studies with a larger number of patients are needed to show the benefit of this technique in patients with diabetic macular edema unresponsive to intravitreal anti-VEGF injections.

## Data Availability

The data used to support the findings of this study are available from the corresponding author upon request.

## Additional Points

*Summary*. The best treatment strategy for refractory DME is not known. Options include switching between anti-VEGF agents, corticosteroids, and vitrectomy. In this study, we describe a new surgical technique for the treatment of refractory nontractional DME. The technique consists of vitrectomy with ILM peeling with a subretinal injection of ranibizumab.

## Supplementary Materials

Supplementary Materials The surgeries were performed using Constellation Vision System 23-gauge system. After core vitrectomy, we stained the ILM with 0.25 mg/mL Coomassie brilliant blue G 250 solution and removed the ILM. We then injected 0.5 mL of filtered air into the subretinal space to detach the fovea, ensuring that the foveal detachment covered the entire area of the DME. This injection of air was performed at the site where the ILM had been removed using a 38-gauge cannula with a pressure of 4 to 6 pounds per square inch. The injected filtered air enters the subretinal space and causes foveal retinal detachment allowing easier subsequent injection of ranibizumab. Subretinal injection of 0.05 ml of ranibizumab (0.5 mg) was done using the same 38-gauge cannula.
